# Conjugation of Meningococcal Lipooligosaccharides Through Their Non-Reducing Terminus Results in Improved Induction a Protective Immune Response

**DOI:** 10.1007/s00005-019-00542-9

**Published:** 2019-04-27

**Authors:** Małgorzata Mieszała, Harold J. Jennings, Marek Drab, Andrzej Gamian

**Affiliations:** 1 Hirszfeld Institute of Immunology and Experimental Therapy, Polish Academy of Sciences, Rudolfa Weigla 12, 53-114, Wrocław, Poland; 2 Institute for Biological Sciences, National Research Council of Canada, Ottawa, ON Canada

**Keywords:** Meningococcal lipooligosaccharides, Conjugate vaccines, Immunological properties

## Abstract

The present studies prove that conjugation of meningococcal lipooligosaccharides through their non-reducing terminus conserves their inner epitopes resulting in conjugates potent to induce a protective immune response. Four different oligosaccharides were obtained by specific degradations of the same L7 lipooligosaccharide (L7-LOS), and each was linked to tetanus toxoid by direct reductive amination. Two were truncated oligosaccharides with incomplete inner epitopes and were obtained by mild acid hydrolysis of lipooligosaccharide. The terminal galactose of one oligosaccharide was additionally enzymatically oxidized. These oligosaccharides were conjugated through a newly exposed terminal Kdo in reducing end or through oxidized galactose localized at non-reducing end of the core, respectively. The third was a full-length oligosaccharide obtained by *O*-deacylation of the L7-LOS and subsequent enzymatic removal of phosphate substituents from its lipid A moiety. The fourth one was also a full-length *O*-deacylated lipooligosaccharide, but treated with galactose oxidase. This allowed direct conjugation to tetanus toxoid through terminal 2-*N*-acyl-2-deoxy-d-glucopyranose or through oxidized galactose, respectively. Comparison of the immune performance of four conjugates in mice revealed, that while each was able to induce significant level of L7-LOS-specific IgG antibody, the conjugates made with the full-length saccharides were able to induce antibodies with increased bactericidal activity against homologous meningococci. Only full-length oligosaccharides were good inhibitors of the binding of L7-LOS to the bactericidal antiserum. Moreover, induction of the significant level of the L7-LOS-specific antibody by full-length lipooligosaccharide conjugated from non-reducing end, provided also the direct evidence that internal core epitopes are fully responsible for the immunorecognition and immunoreactivity.

## Introduction

*Neisseria meningitidis* is a strictly human pathogen which colonizes the upper respiratory tract, usually in the benign form but occasionally converting into one of the fastest human killers. The most dangerous, among the broad array of clinical forms caused by meningococcus is the purulent meningitis and encephalitis, often with bacteremia and septic shock (Verheul et al. 1993). Existing vaccines cover only a minor part of the meningococcus forms, leaving completely lack of protection to the most common form, group B menigococcus. Well studied example of Poland at its geographic crossroads between Europe and Asia, where the group B strains undergo common enrichment, demonstrates the necessity for an universal anti-group B vaccine (Cox et al. 2005; Gryniewicz et al. 2007; Zabicka and Zielinski 2001); the similar situation emerges in many other countries with intensive population’s migrations. Despite the success of current vaccines, based on the most external layer of the pathogen, the capsule, and composed of groups A, C, W-135 and Y capsular polysaccharides, and the improved group C polysaccharide conjugate vaccines (Richmond et al. 2001), the group B polysaccharide remains precluded from the above vaccines, even though it is a major contributor to the burden of disease in developed countries (Peltola et al. 1977; Trotter et al. 2008). This is because of the poor immunogenicity of the group B polysaccharide in both its native (Wyle et al. 1972) and conjugated forms (Devi et al. 1991; Jennings and Lugowski 1981) as compared to the non-B, capsular polysaccharide. Consequently, to generate vaccines aiming covering the complete array of the meningococci, alternative vaccines emerge, based on deeper localized, subcapsular antigens, including lipooligosaccharides (LOS).

The meningococcal LOS have been implicated in the immune response to natural infection (Brandtzaeg et al. 1989; Goldschneider et al. 1969), but their use for direct vaccination is precluded due to their high toxicity. LOS also exhibit considerable antigenic diversity, which presents another major challenge. Currently there are 12 known different immunotypes based on LOS variability (Verheul et al. 1993; Zollinger and Mandrell 1977, 1980), of which types L1–L7 are exclusively associated with groups B and C meningococci, and types L10–L12 with group A meningococci. Only types L8 and L9 overlap between the two groups. The critical epitopes, responsible for the immunotyping, are located in the oligosaccharide (OS) moieties of the LOS (Jakel et al. 2008; Jennings et al. 1984; Mandrell and Zollinger 1977), which have been shown to be structurally diverse (Gamian et al. 1992a; Jennings et al. 1983a; Kogan et al. 1997; Pavliak et al. 1993), as well as having some regions of similarity.

To avoid the toxicity of the LOS, the toxic lipid A moiety can be removed by mild acid hydrolysis, and subsequently the innocuous OS can be conjugated by different methods to protein carriers through their terminal 3-deoxy-d-*manno*-oct-2-ulosonic acid (Kdo) residues (Gu and Tsai 1993; Jennings et al. 1984; Pavliak et al. 1993). Although L10 conjugates (group A meningococci) were able to induce, in mice, OS-specific antibodies that were bactericidal (Gu and Tsai 1993; Jennings et al. 1984), conjugates made with OS associated with groups B and C meningococci in comparison produced antisera with sub-optimal bactericidal activity (Jennings et al. 1984; Pavliak et al. 1993). This was particularly noticeable in the case of the L3 and L7 immunotypes, which are the most prevalent among groups B and C meningococcal isolates (Verheul et al. 1993). L7 immunotype is the desialylated form of L3 (Pavliak et al. 1993), and that is the only difference in the structure of both L3 and L7 immunotypes.

One possible reason for the sub-optimal immune performance of the above conjugates is that the point of cleavage of the LOS, at the Kdo residue, is close to, if not within, the internal OS epitopes, thus impairing their structures. The importance of internal epitopes to the immune response has been documented (Jennings et al. 1987a; Plested et al. 1999) and is due to structural similarity between the non-reducing distal OS chain of the LOS and mammalian tissue antigens, which results in immunodominance of the internal epitopes (Jennings et al. 1983a, 1987a; Mandrell et al. 1988; Tsai and Civin 1991; Verheul et al. 1991). The importance of the internal epitopes was proved by conjugation of LOS through their lipid A terminus what resulted both in the improved immunogenicity of conjugates, and efficacy of vaccines (Mieszala et al. 2003). The recently published trial to conjugate the inner core oligosaccharides of LOS has delivered the conjugate capable of immunizing the animal model but lacking bactericidal activity unless full length de-*O*-acylated lipid A had been used; the core oligosaccharide has been linked via a reducing (proximal) end (Cox et al. 2005). Here, we approached the inner core from the opposite site, the non-reducing (distal) end to be conjugated. Structural similarity of the non-reducing distal OS of the LOS and the tissue antigens causes that this terminal domain is not immunogenic and might be a target of conjugation with protein, without possibly affecting the internal epitope.

To test this hypothesis, we compared the immune response of four tetanus toxoid (TT) conjugates. Two were made with hydrazine-treated, full-length oligosaccharides, linked either terminally (L7-OH,deP) or through oxidized galactose at non-reducing end (L7-OH,ox). Two other conjugates were made with a similarly linked but truncated oligosaccharides, i.e., terminally (L7-OS) or through oxidized galactose at non-reducing end (L7-OS,ox). Conjugation from non-reducing end of full-length LOS did not affect the internal core epitopes, providing also the direct evidence that conserved inner saccharide epitopes are fully responsible for the immunorecognition and immunoreactivity, thus providing the immunogen to target majority of meningococcal groups, for the potentially universal anti-group B vaccine.

## Materials and Methods

### Bacterial Cultivation and Preparation of LOS

Strains M 982B (immunotype L7) and 406Y (immunotype 3) of *N. meningitidis* were grown as described (Mieszala et al. 2003) in Todd-Hewitt Columbia Broth medium (TH-C Broth; Difco, Detroit, MI, USA) at pH 7.3. Ten chocolate agar plates were inoculated with bacteria from a frozen stock or a lyophilized culture and incubated overnight at 37 °C in an atmosphere of 5% CO_2_. The bacteria from the plates were re-suspended in 50 ml of TH-C Broth and transferred to a screw-capped Erlenmeyer flask containing 2 l of TH-C Broth medium. The flask was shaken for 7 h at 37 °C, and the contents were transferred to a 25-l New Brunswick Scientific MFS-128S Microferm fermentor. The bacteria were grown, killed with 1% formaldehyde, and harvested by centrifugation as previously described (Jennings et al. 1983a; Mieszala et al. 2003). Lipooligosaccharides were isolated by a modified phenol-extraction procedure with the use of lysozyme (Gamian et al. 1992a; Johnson and Perry 1976). The crude lipopolysaccharide was purified by fourfold ultracentrifugation for 6 h at 100,000×*g* using a Beckman LE-80 ultracentrifuge.

### Analytical Methods

Solutions were evaporated at reduced pressure below 40 °C in a rotary evaporator. Gel filtration was done on columns (1.6 × 90 cm) of Bio-Gel P4 and P2 (Extra Fine, Bio-Rad Laboratories), using 0.02 M pyridine-acetate buffer (pH 5.4) as eluant at a flow rate of 12 ml/h. A Sephadex G-10 (1.5 × 30 cm, Pharmacia, Sweden) was also employed, using water as the eluant at a flow rate of 24 ml/h. Individual fractions were monitored using a Waters R403 differential refractometer. Conjugates were analyzed for their carbohydrate and protein contents with the phenol–sulfuric acid (Dubois et al. 1951) and bicinchonic acid (Smith et al. 1985) procedures, respectively. Gas–liquid chromatography combined with mass spectrometry (MS) was carried out on Varian Saturn II instrument equipped with a DB-17 capillary column (0.25 mm × 30 m) using ionization potential of 70 eV.

### Chemical Methods

*O*-deacylation of LOS was performed with use of anhydrous hydrazine as previously described (Holst et al. 1993; Pavliak et al. 1993), to obtain LOS-OH. Dephosphorylation of LOS-OH was accomplished by treating with 48% aqueous HF at 4 °C for 48 h. HF was removed with stream of nitrogen and dephosphorylated LOS-OH (LOS-OH,deP_HF_) was purified on Sephadex G-10 column. The core oligosaccharide (OH) was obtained by heating the LOS (10 mg/ml) in 1% acetic acid or 0.1 M sodium acetate buffer (pH 4.2) at 100 °C for 2 h. The insoluble lipid A was removed by centrifugation at 15,000 rpm for 15 min, and the water-soluble OS were separated on Bio-Gel P4 column.

### Enzymatic Oxidation of LOS-OH and OS with Galactose Oxidase

LOS-OH or OS (10 mg) was dissolved in 2 ml 0.02 M phosphate buffer (pH 7.0) and treated with 50 U of galactose oxidase (EC 1.1.3.9, Sigma Laboratories, St. Louis, MO, USA) and 20 U of horseradish peroxidase (EC 1.11.1.7, Sigma Laboratories) (Jennings et al. 1983b) at room temperature for 7 h, then heated at 100 °C for 5 min, centrifuged at 15,000 rpm for 5 min, and water-soluble oxidized material (LOS-OH,ox and OS,ox) was purified on Sephadex G-10 column. Oxidation was monitored by sugar and methylation analysis performed as previously (Mieszala et al. 2003), and position six was deuteriated after oligosaccharides reduction with sodium borodeuteride.

### Treatment of LOS-OH with Neuraminidase and Alkaline Phosphatase

LOS-OH (6 mg) was dissolved in 3 ml of 25 mM sodium acetate (pH 6.8) and treated with 30 mU of neuraminidase (Type VI, Sigma Laboratories) at 37 °C for 3 h. At this time an additional 30 mU of enzyme was added, and the reaction was carried out overnight at 37 °C. The material was filtered through 0.22 μm pore size membrane, then loaded onto a Sep-Pak C18 cartridge (Waters Corporation, Milford, MA, USA) and lyophilized. The product was identified by ^1^H-NMR analysis. Enzymatic dephosphorylation of LOS-OH (10 mg) dissolved in 1 ml of 0.1 M ammonium bicarbonate (pH 8.0) was performed with 70 units of alkaline phosphatase (Boehringer Mannheim or Miles Laboratories, USA) at 56 °C for 24 h as previously described (Mieszala et al. 2003) and obtained LOS-OH,deP was purified on Sephadex G-10 column.

### Coupling of LOS-OH,deP, LOS-OH,ox, OS and OS,ox to TT

LOS derivatives were conjugated to TT using reductive amination procedure previously described (Jennings and Lugowski 1981; Jennings et al. 1984; Mieszala et al. 2003). Briefly, LOS-OH,ox, OS or OS,ox were dissolved in 200 μl of 0.1 M sodium bicarbonate, together with TT monomer and sodium cyanoborohydride (2.5:1:5 w/w/w). The reaction mixture was stirred at 37°C for 4 days, and the progress of conjugation was monitored by a Hewlett-Packard Model 1100 HPLC using a Superose 12 HR 10/30 column (Pharmacia Biotech, USA) with phosphate-buffered saline (PBS) as eluent at a flow rate of 0.4 ml/min. The eluent was monitored using an UV detector operating at OD214 and OD280. The conjugates were purified on a Bio-Gel A 0.5 (Bio-Rad, USA) column (1.6 × 42 cm) eluted with PBS, yielding 5, 4 and 1 mg, respectively. The same procedure was applied to LOS-OH,deP but with use of 0.02 M borate buffer (pH 9.0) as a conjugate solvent, yielding 5 mg of conjugate.

### Immunization Procedure

Groups of 6–8-week-old 10 CF-1 female mice (Charles River, St. Constant, Canada) were injected subcutaneously with each of the conjugates containing 2.5 μg of carbohydrate in saline solution, together with RIBIs complete adjuvant (RIBI Immunochem Research Inc, Hamilton, MT, USA) in a total volume of 0.2 mL. The mice were injected on day 0, 21 and 35, then the antisera were collected on day 45, filtered sterile and stored at − 80 °C.

### Elisa

The experiment was performed as described previously (Gamian et al. 1992b; Johnstone and Thorpe 1982). The 96 wells of microtiter plates (Lynbro/Titertek, No 76-381-04) were coated with solutions of LOS (2 μg/100 μl) in 0.05 M carbonate buffer (pH 9.6) at 37 °C for 3 h and then at 4 °C overnight. The plates were then blocked with 1% bovine serum albumin (BSA) in 20 mM Tris–HCl—50 mM NaCl buffer containing 0.05% Tween 20 (pH 7.5) (T-TBS) at room temperature for 1 h. The contents of the wells were then removed, washed with T-TBS and incubated with serial dilutions of antiserum in PBS (100 μl/well) at room temperature for 2.5 h. After washing with T-TBS, 100 μl of a 1:3000 dilution in PBS of an alkaline phosphatase-labeled goat anti-mouse IgG (H + L, ICN) was added to each well. Following incubation for 1 h at room temperature, the plates were washed with T-TBS (250 μl/well), and 100 μl/well of pNPP substrate (Kirkegaard & Perry Laboratories, Gaithersburg, MD, USA) was added. The reaction was developed for 1 h at room temperature, and the optical densities were read at 410 nm using a Dynatech MR 5000 Microplate Reader.

### ELISA Inhibition

Inhibition of ELISA was performed as previously described (Michon et al. 1991). The 96-well microtiter plate (Lynbro/Titertek, No 76-381-04) was coated with solutions of LOS (2 μg/100 μl) in 0.05 M carbonate buffer pH 9.6, at 37 °C for 3 h and then overnight at 4 °C. The plates were then blocked with 1% BSA in TBS buffer for 1 h at room temperature. Concurrently, a second microtiter plate containing serial twofold dilutions of inhibitors in PBS (50μl total vol.) was mixed with 50 μl of polyclonal serum diluted 1:100 with PBS (or 50 μl of a previously determined dilution of polyclonal serum in PBS that yielded a OD_410_ = 0.8). These mixtures were incubated for 1.5 h at room temperature and the contents of the plate were transferred to the original blocked plate with coated antigen. The plate was then incubated for 2.5 h at room temperature, washed with T-TBS and incubated with alkaline phosphatase-labeled goat anti-mouse IgG (H + L, ICN) diluted 1:3000 with PBS (100 μl/well). Following incubation for 1 h at room temperature and washing with T-TBS, the reaction was developed with pNPP substrate (Kirkegaard & Perry Laboratories) (100 μl/well) at room temperature for 1 h.

### Bactericidal Assay

For bactericidal assay the method of Jennings et al. was employed (Jennings et al. 1987b; Pon et al. 1997). The assay was carried out in tissue culture 96-well polystyrene plates (Costar, No 3595). *N. meningitidis* L7 (strain M982B) was grown overnight on blood agar plates at 37 °C under a 5% CO_2_ atmosphere, followed by inoculating a second plate and incubating it overnight at the same conditions. Twofold dilutions of murine polyclonal antisera were made directly in the plate using HBSS containing 1% casein, diluted to a final volume of 50 μl/well. A suspension of *N. meningitidis* L7 in HBSS-1% casein was made giving an OD_490_ = 0.275 and a final working dilution of bacteria was prepared by a further 1:200 000 dilution. Freshly thawed guinea pig complement was added (20 μl) to each well, followed by 30 μl of the working dilution of bacteria (~ 100 CFU/well). The plate was then placed at 37 °C for 1 h. The contents of each well were mixed before plating (30 μl) on to blood agar plates. The plates were then incubated overnight at 37 °C, 5% CO_2_ and the number of colony-forming units (CFU) were counted. The bactericidal activity of serum (expressed as the percent of bacterial killing by the serum) was calculated relative to the mean value of HBSS buffer/bacteria/complement control well in the following manner: percentage of killing = (CFU_control_ − CFU_serum_/CFU_control_) × 100.

### Immunoglobulin Isotyping

For determination of isotypes, wells of microtitration plate (Lynbro/Titertek) were coated with *N. meningitidis* strain L7 LOS in 0.05 M carbonate buffer pH 9.6 (2 μg/100 μl/well) at 37 °C for 3 h and then at 4 °C overnight. The plate was then blocked with 1% BSA in T-TBS (250 μl/well) at room temperature for 1 h. After threefold washing with T-TBS (250 μl/well), the plate was incubated for 2.5 h at room temperature with murine antisera (100 μl/well) diluted 1:100 with PBS. The plate was then washed five times with T-TBS (250 μl/well), followed by incubating for 1 h at room temperature with rabbit anti-mouse IgG (Bio-Rad, USA) subclass (IgG1, IgG2a, IgG2b, IgG3, IgM, IgA) specific antiserum (100 μl/well). After washing with T-TBS, 100 μl of 1:3000 dilution with PBS of a horseradish peroxidase-labeled goat anti-rabbit IgG (H + L chains, Kirkegaard & Perry Laboratories, USA) was added to each well. Following the incubation for 1 h at room temperature the plate was washed with T-TBS and TMB peroxidase substrate (Kirkegaard & Perry Laboratories) was added (100 μl/well). The reaction was developed at room temperature and stopped with 1 M H_3_PO_4_ (50 μl/well). Optical densities were measured at 450 nm using Dynatech MR 5000 microplate reader.

### Scanning Electron Microscopy

Scanning electron microscopy (SEM) of meningococci was performed at low accelerating voltage of the primary beam with or without coating of the samples, as described in previous work (Drab et al. 2016). Bacteria were applied onto glass coverslips and allowed to adhere for 30 min. The samples were fixed with 2.5% glutaraldehyde in 0.1 M cacodylate buffer at 4 °C for 30 min, then washed in water and dehydrated in series of methanol solutions (25%–50%–75%–100%–100%) in 1-h steps at 4 °C. Samples underwent critical point drying with methanol exchanged for liquid CO_2_ in an automatized approach (CPD300 AUTO, Leica Microsystems, Austria) and imaged with cross-beam scanning electron microscope equipped with Schottky field-emission cathode (Auriga 60, Carl Zeiss, Oberkochen, Germany) at 1.2 kV accelerating voltage, thus the imaging was performed within a mode referred to as the low-voltage, field-emission scanning electron microscopy (LV-FESEM) of non-labeled, critical point-dried sample. In some of the samples, the gentle milling was applied using focused ion beam (FIB) with gallium ions in an integrated cross-beam instrument Auriga 60, Carl Zeiss, Oberkochen, Germany). Before milling the critical-point dried samples were coated with gold, subsequent platinum layers, and FIB milling applied to expose faces for FESEM imaging in a volume imaging approach. Images represent the Everhart–Thornley or in-lens SE detection directly from the sample surfaces, with or without coating applied.

## Results

### Characterization of Oligosaccharides and Their TT Conjugates

**Fig. 1:**
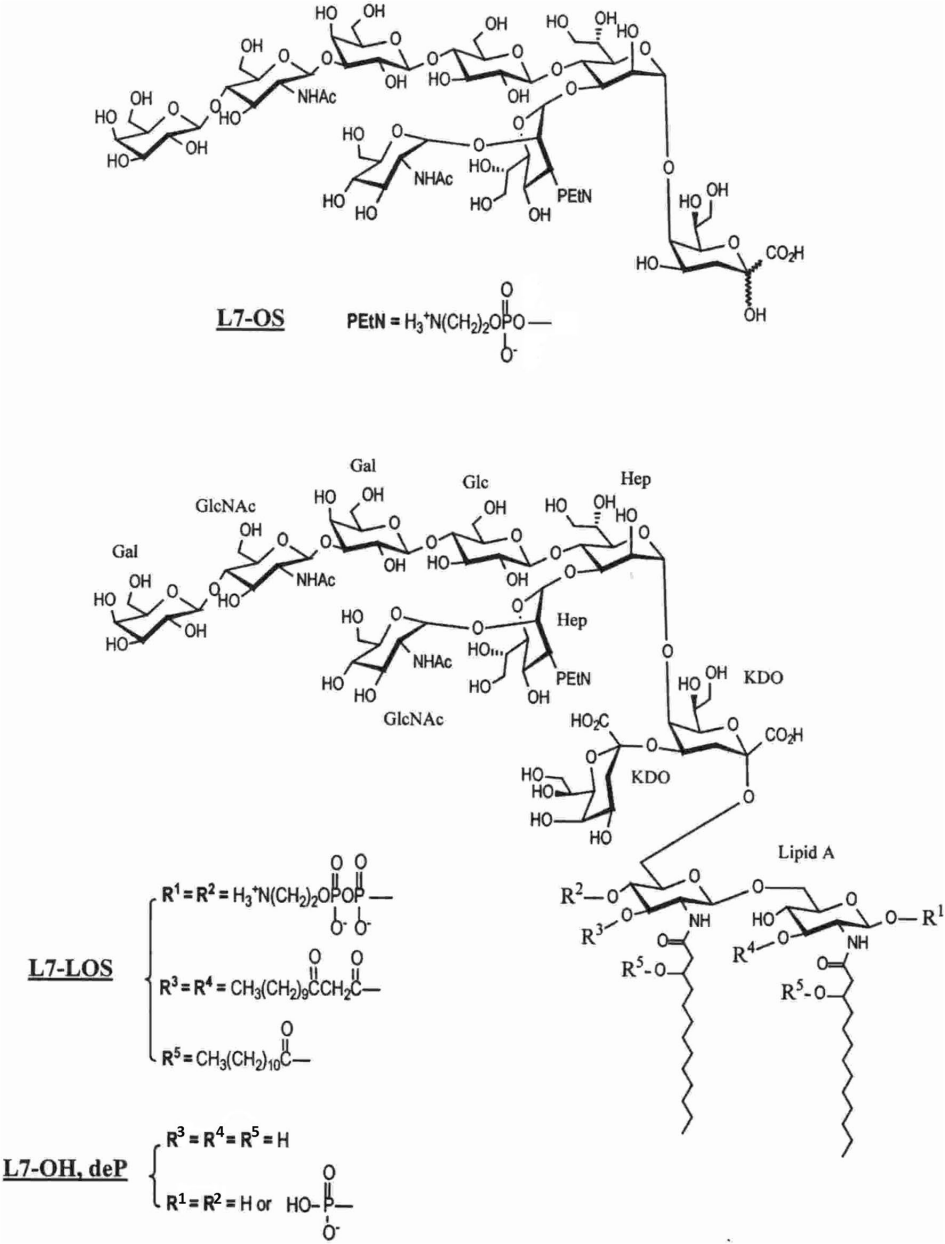
Structures of truncated (L7-OS) and full-length (L7-OH,deP) oligosaccharides of *N. meningitidis* M982B strain. The oligosaccharides with terminal galactose oxidized at C-6 represent structures L7-OS,ox and L7-OH,ox with R^1^ = –PO(OH)_2_ in lipid A domain of latter structure

The L7 oligosaccharide (L7-OS), whose structure is shown in Fig. 1, was prepared as previously (Pavliak et al. 1993) by mild acid hydrolysis of L7-LOS with 1% HOAc and Bio-Gel P4 gel filtration of carbohydrate material. The L7-LOS was also *O*-deacylated using anhydrous hydrazine, then dephosphorylated with alkaline phosphatase to yield L7-OH,deP. The procedures of preparation and structure characterization of enzymatically dephosphorylated L7-LOS (Fig. 1) were described in details previously (Mieszala et al. 2003).

To prepare oligosaccharides with enzymatically oxidized terminal galactose localized at non-reducing end of the core, both truncated L7-OS and *O*-deacylated L7-LOS with anhydrous hydrazine (L7-OH), were treated with galactose oxidase, which resulted in the formation of L7-OS,ox and L7-OH,ox, respectively, ready for conjugation. The terminal galactose in both oligosaccharides was oxidized at position C-6 with formation of aldehyde group and the process was monitored by sugar and methylation analysis. Galactose content was lowered for the terminal galactose which was not detected in methylation analysis after enzyme treatment or was found with six-deuteriated position after oligosaccharides reduction with sodium borodeuteride. Oxidation of terminal galactose was completed in 7 h. The mild procedure of enzymatic oxidation did not introduce structural changes to the core oligosaccharides. This allowed direct conjugation by reductive amination to TT through terminal oxidized galactose residue.

These oligosaccharides were conjugated through a newly exposed terminal Kdo residue in reducing end or through oxidized galactose localized at non-reducing end of the core, respectively, by direct reductive amination procedure previously described (Jennings and Lugowski 1981) and slightly modified (Mieszala et al. 2003). Four conjugates based on *N. meningitidis* L7-LOS (Fig. 1) were prepared to investigate the contribution of conserved inner saccharide epitopes to the induction of protective antibodies directed against pathogenic *N. meningitidis*. Chemical and enzymatic degradations allowed to conjugate the partially detoxified LOS to the TT protein carrier (Mieszala et al. 2003). Conjugation was monitored by HPLC as well as the purification and stoichiometric analysis of conjugates were performed as previously (Mieszala et al. 2003), which proved homogeneity of gal oxidation products not in mixture with conjugates linked through either end. Coupling of the protein to non-reducing end of galactose oxidized LOS-OH and OS modified the external saccharide domains, what allowed to elucidate the degree of contribution of the inner core epitopes to the induction of immune response to LOS.

### Immunogenicity of Conjugates and Bactericidal Activity of Antisera

**Fig. 2:**
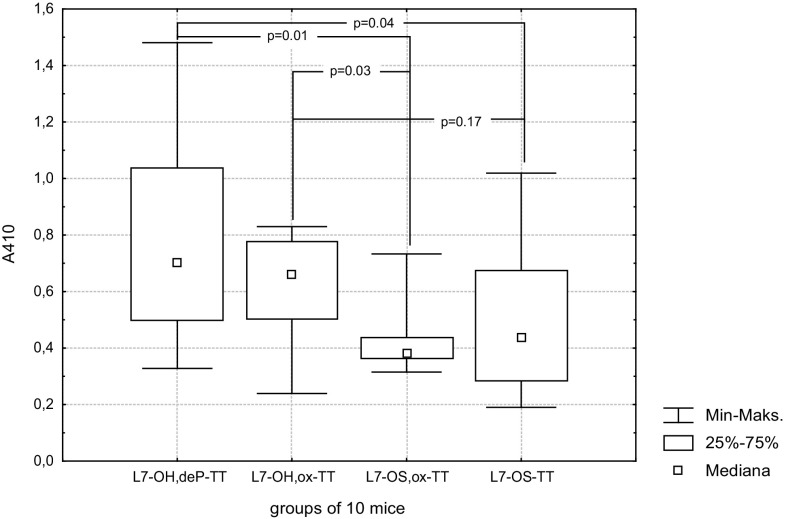
ELISA titers for groups of individual mouse antisera (dilution 1:100) following immunization with L7-OH,de P-TT, L7-OH,ox-TT, L7-OS,ox-TT or L7-OS-TT conjugates. Differences with *p *< 0.05 were considered statistically significant (Mann–Whitney *U* test)

The L7-OH,ox-TT, L7-OS,ox-TT conjugates and comparatively L7-OH,deP-TT and L7-OS-TT conjugates were evaluated for their immunogenicity in mice. Each group of mice was given three subcutaneous injections containing 2.5 μg of carbohydrate together with RIBIs adjuvant before being bled. The ELISA titers of the groups of mice sera (Fig. 2), using the L7-LOS as the coating antigen, showed that all studied conjugates induced L7-LOS—specific antibodies but the level induced by L7-OH conjugates was significantly higher than those induced by L7-OS ones. The L7-OH,deP-TT was noticeably but not statistically significantly more immunogenic than L7-OH,ox-TT conjugate. These facts indicate that both external and internal core parts are involved in induction of immunological response. In addition the subclass distribution of antibodies elicited by four conjugates was not significantly different (Table 1). All conjugates were able to produce high levels of potentially bactericidal IgG2a, IgG2b and IgG3 antibodies.

**Table 1:** Immunoglobulin isotype distribution of L7-OH,deP-TT, L7-OH,ox-TT, L7-OS,ox-TT and L7-OS-TT antisera

Subclass	L7-OH,deP-TT (%)	L7-OH,ox-TT (%)	L7-OS,ox-TT (%)	L7-OS-TT (%)
IgG1	9.1	18.6	15.3	18.0
IgG2a	18.1	15.4	14.0	18.4
IgG2b	25.1	18.3	19.3	15.3
IgG3	9.1	13.8	14.3	16.6
IgM	24.7	19.0	19.9	16.6
IgA	13.9	14.9	17.2	15.1

**Fig. 3:**
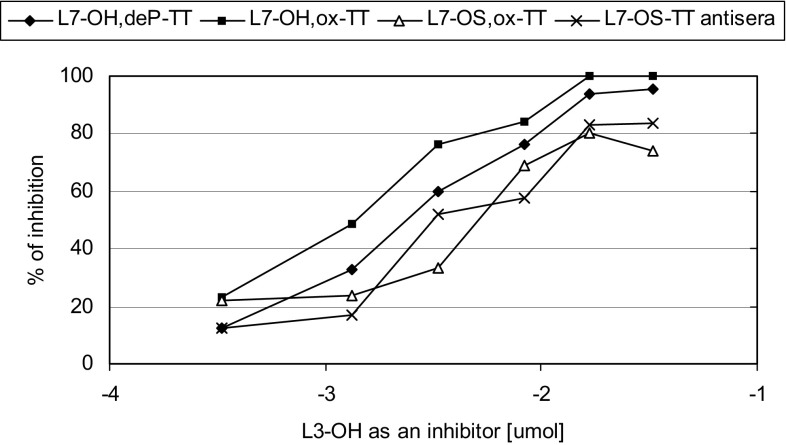
Oligosaccharide inhibition by L3-OH of the binding to coated L3-LOS of sera against L7-OH,deP-TT and L7-OH,ox-TT, L7-OS,ox-TT and L7-OS-TT conjugates

**Fig. 4:**
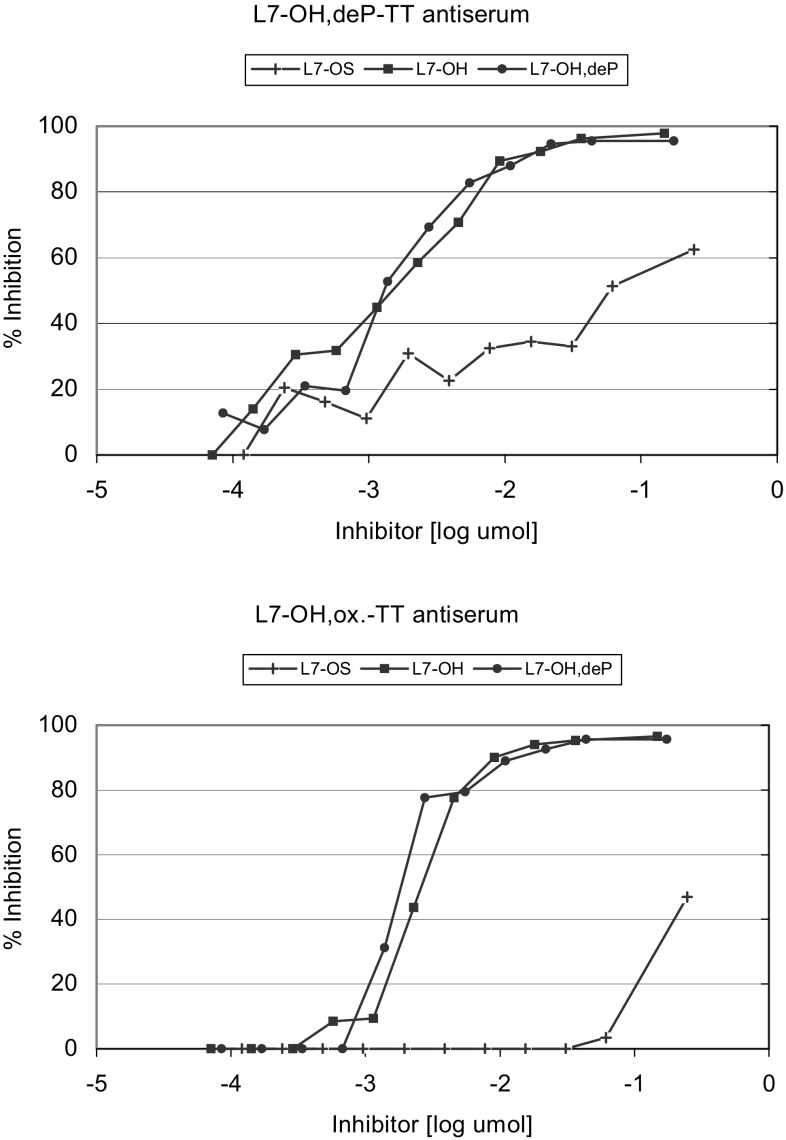
Oligosaccharide inhibition of the binding of L7-LOS to sera against L7-OH,deP-TT and L7-OH,ox-TT conjugates

To characterize the murine polyclonal sera directed against L7-LOS conjugates a competitive inhibition ELISA was performed using L3-LOS (Fig. 3) or L7-LOS (Fig. 4) as coating antigens. The L3-LOS has the same structure as the L7-LOS except it is terminally sialylated (Pavliak et al. 1993). This substitution may result in the modifying of the external terminal Gal-containing saccharide epitope like in L7-OH,ox-TT and L7-OS,ox-TT conjugates. Indeed, the L3-OH with sialylated terminal galactose appeared to be a better inhibitor of L7-OH,ox-TT antiserum than L7-OH,deP-TT antiserum (Fig. 3). Comparison of the reactivity of the four sera, when L3-OH was used as an inhibitor (Fig. 3), showed higher LOS—specificity of the conjugates made with the full-length LOS than the truncated oligosaccharides. For further characterization of the full-length LOS antisera the system with L7-LOS as a coating antigen and L7-OS as inhibitors was applied (Fig. 4). Visual inspection of the curves obtained using the L7-LOS oligosaccharides revealed that both L7-OH and L7-OH,deP were equivalent and good inhibitors, whereas L7-OS was a poor inhibitor. It was particularly seen when the L7-OH,ox-TT antiserum was examined. This corroborates result from ELISA experiment (Fig. 2) indicating that both external and internal core parts of L7-OH,deP-TT are involved in induction of immunological response.

**Fig. 5:**
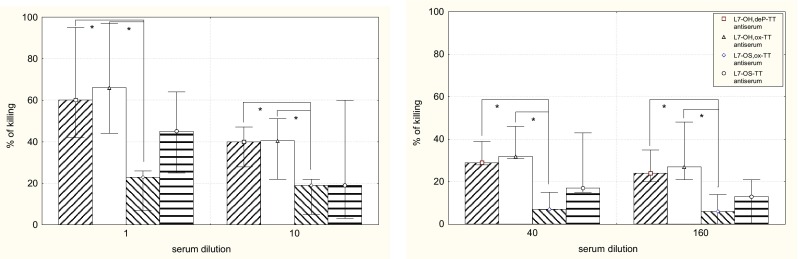
Bactericidal activity of the pooled antisera of the mice immunized with L7-OH,deP-TT, L7-OH,ox-TT, L7-OS,ox-TT or L7-OS-TT conjugates. The bar graph represents the results of three different experiments each made in duplicate. No bactericidal activity was observed in experiments carried out with complement but without antibody and in experiment with antibody but without complement. Differences with *p *< 0.05 were considered statistically significant (Mann–Whitney *U* test). Statistically significant correlations are marked with stars

The bactericidal activities of the antisera induced in mice by L7-OH,deP-TT, L7-OH,ox-TT, L7-OS,ox-TT and L7-OS-TT conjugates against the homologous immunotype organism are shown in Fig. 5. The difference in the ability of four conjugates to induce bactericidal antibodies is surprising, because all conjugates were able to produce L7-LOS—binding antibodies with isotypes, which at least had the potential of being bactericidal (Table 1). We notified that the antisera induced by full-length LOS conjugates had greater bactericidal activity (Fig. 5), which was statistically significant in comparison with that induced by L7-OS,ox-TT. This observation as well as the inhibition experiments provided evidence that important bactericidal epitopes are lost when LOS is subjected to mild acid hydrolysis.

**Fig. 6:**
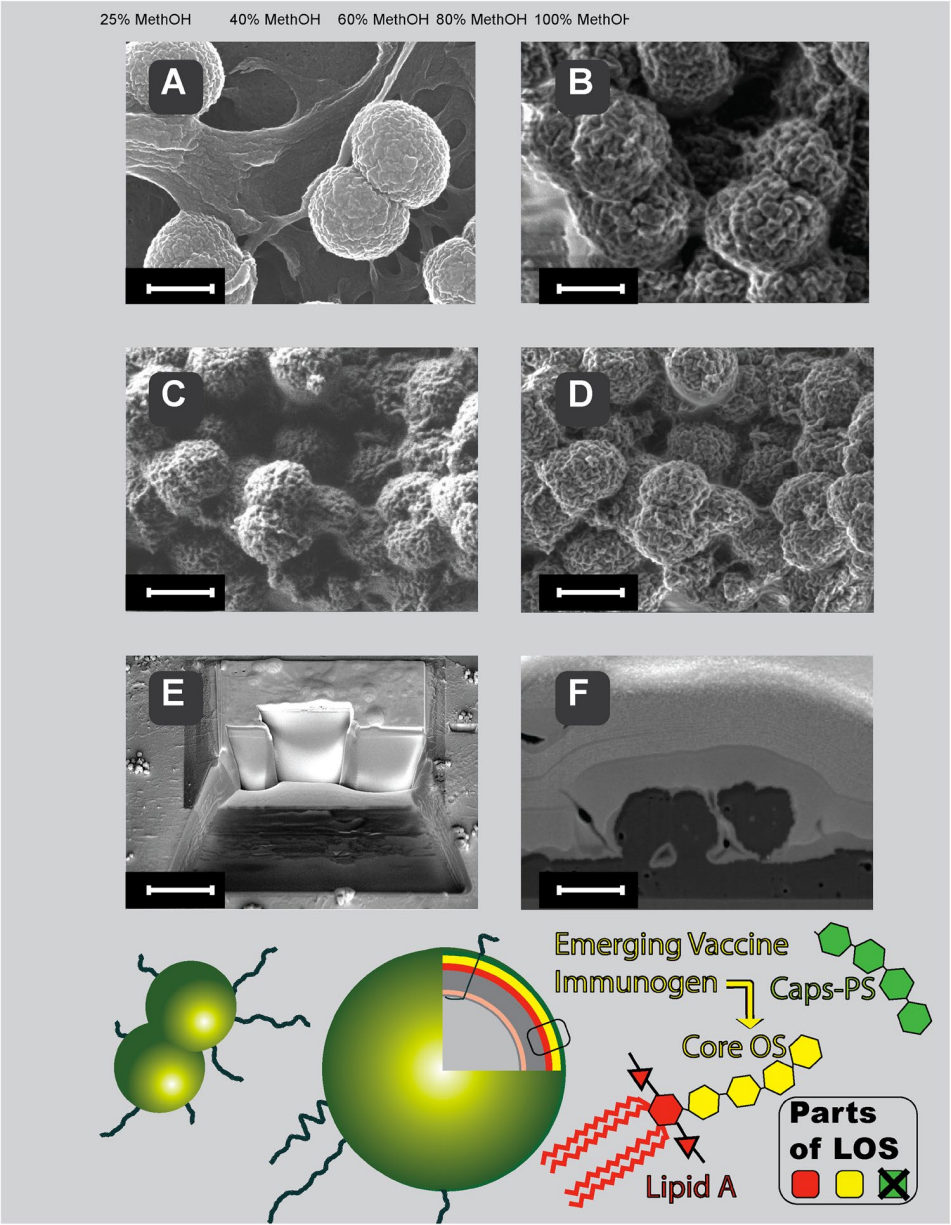
Localization and structure of LOS in meningococci (bottom panel) and SEM imaging (top panel) of the meningococci coated (**a**), non-coated (**b**–**d**) and cross-sectioned by gentle FIB milling (**e** and **f**). Lipooligosaccharide (LOS) of *N. meningitidis* does not contain a long polysaccharide chain as normally see in other Gram-negative bacteria lipopolysaccharide. We took advantage of this LOS structure feature that enables better penetration of antibodies to the core oligosaccharide and efficient microbe killing properties of the proposed vaccine strategy. Please notice that high lateral resolution in classical high-voltage SEM in platinum-coated samples (heavy-metal elements coat) is comparable to the high lateral resolution in emerging low-voltage SEM in non-coated samples (light bio-elements only)—native chemical composition only. **a** Platinum-coated classical mode of *N. meningitidis* imaging (acceleration voltage 20 keV). *Everhart*–*Thornley* SE2 electrons detector. Scale-bar = 600 nm. **b** Non-coated mode of *N. meningitidis* imaging at low energies (acceleration voltage 1 keV). *Everhart*–*Thornley* SE2 electrons detector. Scale-bar = 600 nm. **c** Non-coated mode of *N. meningitidis* imaging at low energies (acceleration voltage 1 keV). *Everhart*–*Thornley* SE2 electrons detector. Scale-bar = 1.2 μm. **d** Non-coated mode of *N. meningitidis* imaging at low energies (acceleration voltage 1 keV). In-lens SE1 electrons-detector. Scale-bar = 1.2 μm. **e** FIB-milling of critical-point dried *N. meningitidis* deposited on glass coverslip. Coating with thick layer of gold and platinum allows smooth cross-section face to be serially milled and further analyzed with *Everhart*–*Thornley* SE2 electrons detector. Scale-bar = 10 μm. **f** Face of cross-sectioned *N. meningitidis* exposed by gentle FIB milling and imaged with low-energy SEM using energy-selective back-scattered electron detector (EsB electrons-detector. The EsB detector allows for elemental analysis when desired, based on Auger transitions detected (Drab et al. 2016; Drab 2018) (scale-bar = 1 μm)

Scanning electron microscopy (Fig. 6) of meningococci performed at low accelerating voltage of the primary beam with and without coating of the samples, shows the cell surface. The progress in the SEM imaging of the *N. meningitidis* bacterial cell is depicted in Fig. 6 top panels. Classical coating of meningococci with platinum (Fig. 6a) for scanning electron microscopy has been recently supplemented by coating-free imaging that is compatible with chemical mapping of bacterial surface (Fig. 6b–d). LV-FESEM is useful in imaging of meningococci also when combined with deep gentle milling of bacteria with focused ion beam, as we show in Fig. 6e, f.

## Discussion

In this work, we demonstrate that conjugation from non-reducing end of full-length LOS did not affect the conserved internal core epitopes, which participates in the induction of immunological response and we proved the bactericidal properties of the vaccine based on such conjugate. Our study delivers a novel vaccine strategy directed against group B of *N. meningitidis*, the most frequent clinical cause of meningitis, so far not targeted by available vaccines. Thus, we fill the existing gap in the vaccination spectrum against deadly pathogen *N. meningitidis*.

The majority of cases of meningococcal infections in Europe are due to serogroup B and C. Although the overall yearly incidence for meningococcal disease is not high (2.26 per 100,000 inhabitants), 64.9% of meningococcal meningitis cases is in children up to 4 years old. While serogroup B strongly dominates to over 90%, an increasing number of cases of infections caused by serogroup C is observed nowadays (Zabicka and Zielinski 2001). The effective and safe vaccines against meningococci are thus still emerging global problem. It has been already postulated that meningococcal LOS core oligosaccharide—protein conjugate may be candidate vaccine to prevent meningitis caused by meningococci. The scheme of LOS localization and core OS structure in *N. meningitidis* is shown in Fig. 6 (bottom). Due to advances in LV-FESEM the quality of imaging of bacteria remains high despite the lack of metal or carbon coating. Native bio-elements of *N. meningitidis* were capable to generate contrast despite they belong to the top three periods of Mendeleev table, normally not useful for electron microscopy due to too low atomic number (Z) and traditionally recognized as of too low electron density for SEM. The key progress for direct imaging of non-coated cells with SEM was done by introducing low-energy imaging mode, with incident beam of electrons having energies at the level of 1 keV. Such low energy of electrons allows for efficient interaction with shallow surface volume and yields sufficient quanta of single non-elastic interactions with biosample and back-scattered electrons, containing information on chemical composition (Drab et al. 2016). From practical point of view the LV-FESEM approach generates better lateral resolution on native bio-surfaces than classical SEM; in addition chemical mapping can be performed thanks to lack coating, as described before (Drab 2018; Drab et al. 2016). Bacteria coated with metal (Au and Pt) layer can be made accessible for LV-FESEM by serial milling with gallium ions which expose surfaces of cross-sections that can be serially imaged and chemically mapped when desired. Thus, not only better resolution of the native surface became possible but also volume imaging and three-dimensional reconstruction of biological samples both in topography (Fig. 6e, f) and chemical elemental mapping in 3D by low-voltage SEM (Drab 2018).

It has been shown that inner core epitopes of *N. meningitidis* LOS are conserved and accessible to antibody hence potent as a vaccine candidate (Plested et al. 1999). Moreover, it has been demonstrated that both bactericidal and opsonophagocytic antibodies can be generated against these inner core epitopes of *N. meningitidis* (Mackinnon et al. 2002). Remarkably there were no effects observed of the capsule on the accessibility of monoclonal anti-inner core antibody (Mab B5), as anti-capsular and anti-core antibodies were co-localized in wild-type cells grown in vitro and in vivo (Plested et al. 2003). Convalescent sera from meningococcal infection and also non immunized human sera possess antibodies specific to meningococcal LOS and its inner core structures suggesting the potential use of LOS as a vaccine (Andersen et al. 2002; Jakel et al. 2008; Monteiro et al. 2003). In previous study on LOS conjugate vaccine, the full length OS obtained by *O*-deacylation of the L7 LOS was dephosphorylated at lipid A moiety by alkaline phosphatase and directly conjugated to protein. Such conjugate was able to induce significant level of bactericidal antibodies against homologous meningococci. This approach also proved the importance of conserved inner saccharide epitopes to the immune performance of meningococcal LOS-protein conjugate vaccine (Mieszala et al. 2003). Detoxifying LOS by mild alkaline *O*-deacylation and exposing the terminal reducing glucosamine residue to create the distal linkage point allowed to protect the inner epitopes. The L3 and L7 immunotypes are the most prevalent found among group B *N. meningitidis* disease isolates and the LOS of group B and C have an immunosuppressive lacto-*N*-neotetraose chain of poor immunogenicity. The L3 and L7 immunotypes are closely related, differing only by the addition of terminal sialic acid to the lacto-*N*-neotetraose chain of the former. Antibodies against lacto-*N*-neotetraose domain and its sialylated form are not desired to be induced as they might cross-react with host tissue. Nor the presence or absence of sialic acid has an effect on the inner epitope, since both monoclonal antibodies B5-positive and negative strains had high sialylation states as shown in gels and electrospray-ionization MS (Plested et al. 1999). The autoantibody are formed during *Campylobacter jejuni* infections and considered to be then involved in interactions with tissue gangliosides in Guillain–Barre neurological disorder (Andersen et al. 2002). Thus, it has been postulated by others the necessity for meningococcal LOS to remove the outer-core region, which express glycans homologous to human blood-group antigens, as a required first step to avoid undesirable immunological reactions following vaccination. Here we show, that instead of removal, the non-reducing end of full-length saccharide might be blocked or used as a linkage point for conjugation to protein located distally from otherwise non-altered epitope. To test this hypothesis, we conjugated to TT an L7-OS having conserved inner epitopes via the non-reducing end, and compared the immune properties of the conjugates mainly to those of a TT conjugates made as previously described which linked to reducing end (Mieszala et al. 2003). Conjugates made with truncated oligosaccharides, in which the inner epitopes were incomplete, were of low immunogenicity. We induced significant levels of the L7-LOS-specific IgG antibody by full-length LOS conjugated from non-reducing end, comparable to that induced by full-length LOS conjugated from reducing end. Both conjugates made with the full-length saccharides were able to induce significant level of bactericidal activity against homologous meningococci. The observations that the L7-OH,deP-TT was slightly more immunogenic than L7-OH,ox-TT conjugate (Fig. 2) and L7-OS was better inhibitor of L7-OH,deP-TT antiserum than L7-OH,ox-TT antiserum (Fig. 4) indicate that both external and internal core parts of L7-OH,deP-TT conjugate are involved in induction of immunological response. In contrast, L7-OH,ox-TT induce only anti-internal core part antibodies, which, most relevantly, expressed slightly higher level of bactericidal activity. These results indicate that uncovered external part of core in certain degree participates in the induction of immunological response. Blocking the external core part in L7-OH,ox-TT permits induction of only immunological response to internal core, thus avoiding formation of potentially harmful antibodies. This approach may be considered as an effective strategy to avoid induction of auto-antibodies and still capable to comprise *Neisseria, Haemophilus* and other species with terminal galactose at non-reducing end with the epitope localized in the inner part of core of endotoxin. Conjugation through non-reducing end has a potential to fill the gap in the vaccines’ armory against meningitis.
